# Sex- and Neuropsychiatric-Dependent Circadian Alterations in Daily Voluntary Physical Activity Engagement and Patterns in Aged 3xTg-AD Mice

**DOI:** 10.3390/ijms232213671

**Published:** 2022-11-08

**Authors:** Daniel Alveal-Mellado, Lidia Castillo-Mariqueo, Lydia Giménez-Llort

**Affiliations:** 1 Institut de Neurociències, Universitat Autònoma de Barcelona, 08193 Barcelona, Spain; 2 Department of Psychiatry and Forensic Medicine, School of Medicine, Universitat Autònoma de Barcelona, 08193 Barcelona, Spain

**Keywords:** Alzheimer’s disease, animal model, sex differences, rehabilitation, exercise, physical activity, circadian rhythms

## Abstract

Alzheimer’s disease (AD) patients suffer from circadian rhythm alterations affecting their daily physical activity patterns with less willingness to perform a voluntary exercise. In preclinical studies, there is no clarity on whether animal models of AD can replicate these impairments. Here, we provide a proof of concept of the performance and behavioral effects of four weeks of voluntary wheel running (VWR) in a group of 14-month-old male and female 3xTg-AD mice at advanced stages of AD and the daily variance (behavioral circadian rhythmicity) of VWR associated with sex and their neuropsychiatric-like phenotype. Higher levels of horizontal exploration in the open field (OF) test were found in mice submitted to exercise. A linear mixed effect model showed significant sex-dependent differences in the VWR activity performed on the first night of follow-up, with high-NIBI males running less than high-NIBI females. Thus, an influence of NPS-like symptoms on the circadian patterns of VWR may account for such differences. In addition, males remained more active than females during diurnal periods. We hypothesize that this increment in energy expenditure during resting periods may be related to hyperactive behavior, similar to that observed in humans’ exacerbated agitation or sundowning behavior. These findings support the usage of the 3xTg-AD mouse as a reliable model for studying circadian rhythm alterations in AD and, at the translational level, the importance of tailored and individualized physical activity programs in clinical settings.

## 1. Introduction

An insidious cognitive decline comprising anterograde memory and executive function impairments are the main diagnostic criteria for dementia caused by Alzheimer’s disease (AD) [1]. In addition, a broad spectrum of neuropsychiatric symptoms (NPS) may accompany the instauration of the disease [2], including affective disorders (anxiety and depression), behavioral and emotional difficulties (apathy and mood fluctuation), psychotic symptoms (hallucinations and delusions), and sundowning behavior (circadian rhythm alterations) [3]. Indeed, when severe, such dysfunctions represent the leading cause of institutionalization [4,5]. Because of the heterogeneity of the clinical phenomena, NPS are also referred to as behavioral and psychological symptoms of dementia (BPSD) [6,7].

Circadian rhythm dysfunctions (CRD) are also reported in the early stages of the disease, and it comprises sleep, thermoregulation, and movement disorders [8]. CRD are also observed in the healthy aging process due to changes in afferent pathways to the circadian pacemaker of the organism, the suprachiasmatic nucleus (SNC) [9]. However, AD patients suffer more pronounced changes, and degeneration affects the SCN even before the cognitive decline begins [10]. Furthermore, CRD have been proposed as a preclinical sign of the disease [11]. Therefore, nonpharmacological strategies that can be implemented in individuals at risk or be part of the individuals’ lifestyle can play an important role as prevention strategies during asymptomatic and prodromal stages to hamper or counteract the underlying detrimental processes.

Environmental factors such as maintaining moderate levels of physical activity (PA) may prevent cognitive decline [12,13] and modify neuropathological changes that occur in the early stages of AD [14]. However, clinical evidence warns that NPS/BPSD and CRD negatively influence routine exercise and physical rehabilitation engagement in these patients [15,16]. Thus, low levels of PA have been reported in free-living and institutionalized AD patients, with a marked reduction in women [16,17,18]. At the translational level, the effects of PA have also been studied in animal models of AD. Thus, improvements in cognitive and BPSD-like symptoms are reported at the early and moderate stages of the disease [19,20], whereas the effects of PA at more advanced stages remain unclear. We have also shown that, despite the benefits, a forced exercise paradigm may also exert some minor adverse effects on females [21]. In the present work, we addressed the animal’s engagement in the voluntary wheel-running paradigm for the first time.

Equally important have been the study of CRD in aging and AD using rodent models. Daily movement activity variations, measured as “in cage” activity or running wheel (RW) activity, are frequently reported with a general age-dependent decline [21] and greater levels of PA in females [22,23], probably attributable to a protector effect of estrous cycle hormones [24].

The triple transgenic model for AD (3xTg-AD) developed by Frank LaFerla’s laboratory [25] has shown good face and construct validity [26], replicating the extracellular beta-amyloid plaques and intracellular neurofibrillary tangles accumulation within specific brain areas, such as the hippocampus and neocortex, considered the neuropathological hallmarks of the disease [25,27,28].

Cognitive symptoms arise in animals’ early ages and can be detected in tests evaluating spatial orientation and working memory (i.e., Morris water maze and T-maze) [29]. On the other side, tests involving novel environments (i.e., corner test and open-field test) have shown sensible to detect noncognitive symptoms (NPS-like symptoms) through the induction of higher freezing behavior and reduced horizontal exploration. These findings have been previously referred to as novelty-induced behavioral inhibition (NIBI) [30,31].

Regarding CRD, the model exhibits circadian alterations [29] and reproduces sleep and movement activity disorders at 6 months old [32,33]. Nevertheless, the temporal progression of such impairments at older ages has not been fully depicted.

The present work aims were (1) to model the pattern of behavioral circadian rhythmicity of PA in a group of 3xTg-AD animals at advanced ages submitted to daily voluntary wheel running (VWR) and (2) to evaluate the effects of 30 days of PA in noncognitive symptoms of AD.

## 2. Results

**Figure 1:**
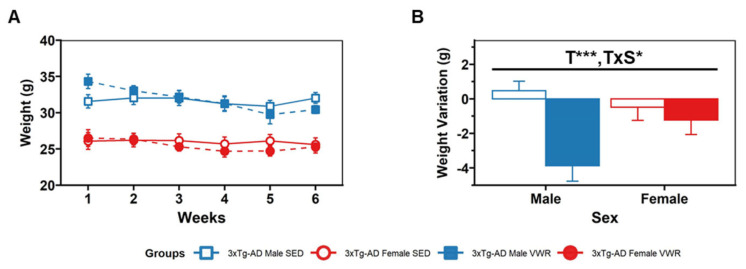
Body weight (**A**) at baseline (week 1) and after 30 days follow-up (week 6). The intervention took place between weeks 2 and 5. (**B**) Weight variation per group (weight at week 6 -week 1). Factorial analysis: D, days; T, treatment; S, sex. * *p* < 0.05; *** *p* < 0.001.

Body weight was monitored from the day before the start of the behavioral battery of tests until the week after the end of the 30-day follow-up (see Figure 1). Weight variation differences were analyzed through a 2 × 2 × 2 (sex × treatment × days) ANOVA test showing a treatment effect [T, F(1,30) = 11.325, *p* = 0.002] with a significant decrease in weight after 30 days in the VWR group [SED vs VWR, t(18,16) = 2.97, *p* = 0.006, *t*-test], mostly due to statistically significant differences observed in males [SED males vs VWR males, t(10,7) = 4.16, *p* = 0.001, *t*-test]. Indeed, the sex × treatment interaction effect [F(1,30) = 5.70, *p* = 0.023] shows that SED females also showeda negative weight variation.

### 2.1. Corner (CT) and Open Field (OF) Tests

**Figure 2:**
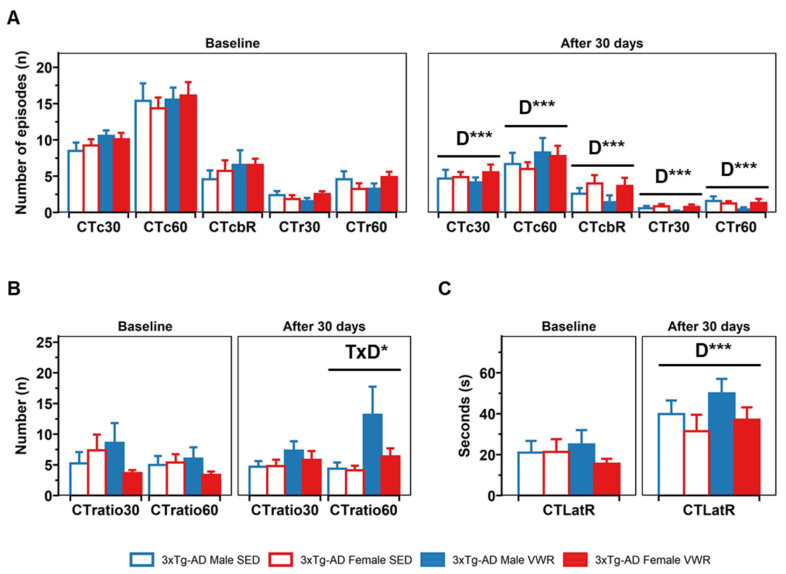
Corner test. (**A**) Horizontal activity: number of corners visited at 30 s (CTc30) and 60 s (CTc60), number of corners before rearing (CTcbR), rearing at 30 s (CTr30) and 60 s (CTr60) (**B**) ratios of horizontal/vertical activity: corners visited/n rearings at 30 s (CTratio30) and 60 s (CTratio60) and (**C**) vertical activity: rearing latency (CTLatR). Factorial analysis: D, days; T, treatment; S, sex. * *p* < 0.05; *** *p* < 0.001.

In the CT, no baseline differences between groups were found (Figure 2). By contrast, a 2 × 2 × 2 (sex × treatment × days) mixed (split-plot) ANOVA test yielded a days effect in almost all variables evaluated (D, all Fs(1,30) > 14.09, *p* < 0.001), indicating a reduction in exploratory activity and an increment in neophobia in the pre-post analysis. Thus, all animals (n = 34) reduced the number of corners at 30 s (t(34) = 6.35, *p* < 0.001, paired *t*-test) and 60 s (t(34) = 6.51, *p* < 0.001, paired *t*-test); the number of rearings at 30 s (t(34) = 5.94, *p* < 0.001, paired *t*-test) and 60 s (t(34) = 5.62, *p* < 0.001, paired *t*-test), and the number of corners until first rearing (t(34) = 3.65, *p* < 0.001, paired *t*-test). Conversely, an increase in the latency of first rearing when re-tested was found (t(34) = −4.44, *p* < 0.001, paired *t*-test). In addition, a treatment × days interaction effect was observed in the ratio of visited corners/rearings in 60 s (CTratio60) (T × D, F(1,30) = 7.14, *p* = 0.012) with an increase in the VWR animals (re-test vs. baseline, t(16) = −2.51, *p* = 0.024, paired *t*-test) and higher values compared with the SED group (t(18,16) = −2.21, *p* = 0.041, *t*-test).

**Figure 3:**
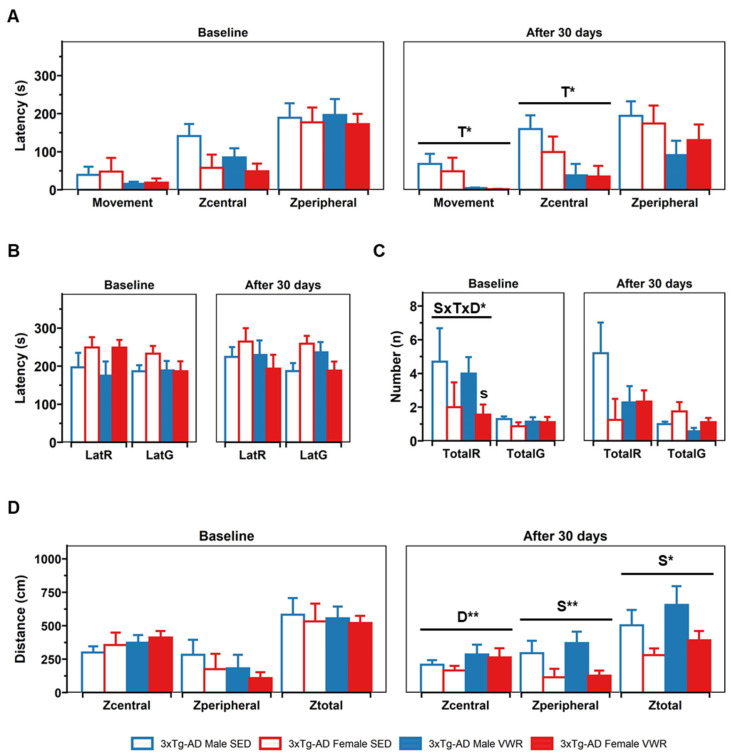
Open field test. (**A**) Horizontal activity latencies: movement latency (movement), latency to start exploring in the central zone (Zcentral), latency to reach the periphery (Zperiphery). (**B**) and (**C**). Vertical activity: rearing latency (LatR), grooming latency (LatG), total rearing episodes in 5 min (TotalR), total grooming episodes in 5 min (TotalG) (**D**) distance covered in the OF in the central zone (Zcentral), peripheral zone (Zperiphery), and total field (Ztotal). Factorial analysis: D, days; T, treatment; S, sex; s < 0.05 vs. male counterpart in the same treatment group. * *p* < 0.05; ** *p* < 0.01.

In the OF test, a 2 × 2 (sex × treatment) ANOVA showed a treatment effect after 30 days in the latency of movement (movement) [T, F(1,30) = 5.72, *p* = 0.023] and in the latency to start exploring the central zone (Zcentral) [T, F(1,30) = 7.25, *p* = 0.011] (Figure 3). A post hoc analysis yielded a reduction of these parameters in the group submitted to VWR compared with SED animals [VWR vs. SED, all t(16,18) > 56.6, *p* < 0.015, *t*-test]. Regarding vertical activity, a sex × treatment × days interaction was found [S × T × D, F(1,30) = 5.07, *p* = 0.032, Mixed ANOVA]. A further analysis showed that significance could be explained by sex differences in the VWR group at baseline [3xTg-AD male VWR vs. 3xTg-AD female VWR, t(7,9) = 2.4, *p* = 0.042]. No differences were found in the latency of rearing, the latency of grooming, or emotionality (urination and defecation boli) (data not shown). Finally, when the distance covered by the animals was analyzed, a days effect was found in the central zone [D, F(1,30) = 12.60, *p* = 0.001] with a decrease in all groups when retested [retest vs. baseline, t(34) = 3.66, *p* < 0.001, paired *t*-test]. Moreover, sex effects were found in the periphery [S, F(1,30) = 7.98, *p* = 0.008] and total field [S, F(1,30) = 6.02, *p* = 0.02] after 30 days with males covering more distance.

### 2.2. T-Maze Spontaneous Alternation (TMSA)

**Figure 4:**
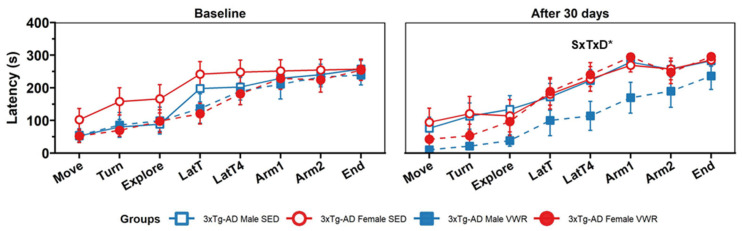
TMSA test at baseline (**left**) and after 30 days follow-up (**right**). Latency to move (Move), latency to turn (Turn), latency to reach the maze intersection (LatT) and cross the intersection with the four paws (LatT4), latency to explore the first (Arm1) and second (Arm2) arms and latency to complete the test completion criteria (End). Factorial analysis: D, days; T, treatment; S, sex. * *p* < 0.05.

The TMSA (Figure 4 test showed no differences in the latencies related to freezing behavior (to move and turn) and exploration; namely, the time until start to explore (Explore), reach the intersection (LatT), and the time spent until reaching the first (Arm1) and second (Arm2) arm of the maze, and the test completion criteria (End) [All Fs(1,29) < 3.49, *p* > 0.05, mixed ANOVA]. However, a sex × treatment × days interaction was found in the time elapsed until crossing the intersection with their four paws (LatT4) [F(1,29) = 4.80, *p* = 0.037, mixed ANOVA] after 30 days, with 3xTg-AD males VWR reaching this goal in a shorter time compared with 3xTg-AD females VWR [t(7,8) = −2.20, *p* = 0.048, *t*-test]. Moreover, considering the goals in the TMSA ethogram as a whole (pooled data), a trend is observable with better performance of the 3xTg-AD males VWR compared with all other groups [S × T × D, F(1,29) = 3.90, *p* = 0.058] in the retest. Instead, no significant differences were found after 30 days in the number of errors and vertical activity (rearings and grooming) in the test (data not shown).

### 2.3. Sensorimotor Assessment

**Figure 5:**
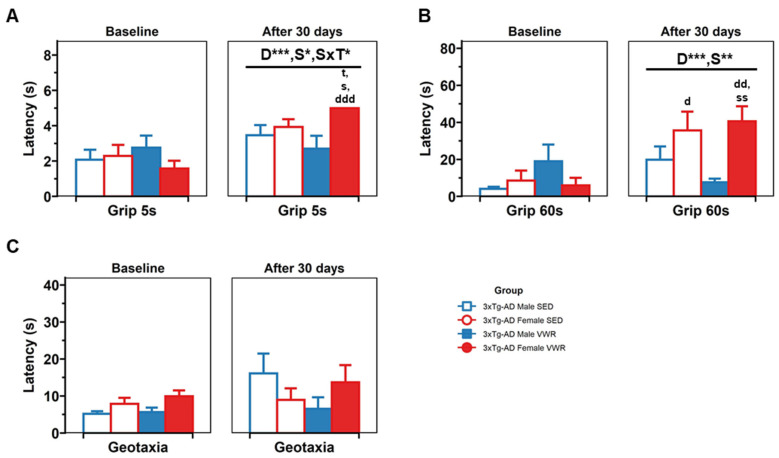
Sensorimotor assessment. (**A**) Muscular strength at baseline and after 30 days. (**B**) Muscular endurance at baseline and after 30 days. (**C**) Geotaxia at baseline and after 30 days. Factorial analysis: D, days; T, treatment; S, sex. t < 0.05. * *p* < 0.05; ** *p* < 0.01; *** *p* < 0.001; d < 0.05, dd < 0.01 and ddd < 0.001 vs. the same group at baseline; s < 0.05 and ss < 0.01 vs. male counterpart in the same treatment group.

The sensorimotor assessment comprised (1) the muscular strength (5 s) and muscular endurance (60 s) grip tests evaluating forelimbs strength and (2) geotaxis, focused on the response to gravity assessment (Figure 5). In the muscular strength trial, a days effect [D, F(1,29) = 19.95, *p* < 0.001], sex × day [S×D, F(1,29) = 5.23, *p* = 0.03] and a sex × treatment × day interaction effects [S × T × D, F(1,29) = 5.57, *p* = 0.025] were found. A post hoc analysis showed that all groups increased their latency after 30 days [t(34) = −4.30, *p* < 0.001, paired *t*-test], females last longer than males after 30 days (3.21 vs. 2.77 s) [t(17,17) = −2.66, *p* = 0.01], and 3xTg-AD VWR females had better performance after 30 days compared with their values at baseline [t(9) = −7.79, *p* < 0.001, paired *t*-test] vs. 3xTg-AD SED females [t(8,9) = −2.42, *p* = 0.046] and vs. 3xTg-AD VWR males [t(7,8) = −3.14, *p* = 0.021]. In the muscular endurance trial a days effect [D, F(1,30) = 13.98, *p* < 0.001 ] and a sex × days interaction effect [F(1,30) = 10.51, *p* = 0.003, mixed ANOVA] were found with females improving their performance when retested [t(17) = −4.78, *p* < 0.001, paired *t*-test] and resisting more than males after 30 days [t(17,17) = −3.06, *p* = 0.005]. In a post hoc, 3xTg-AD SED females and 3xTg-AD VWR females improved after 30 days and differences were found between the 3xTg-AD VWR females and 3xTg-AD VWR males [t(7,9) = −3.92, *p* = 0.003, *t*-test]. Respecting geotaxis, no treatment effect nor differences between groups were found.

### 2.4. Rotarod

**Figure 6:**
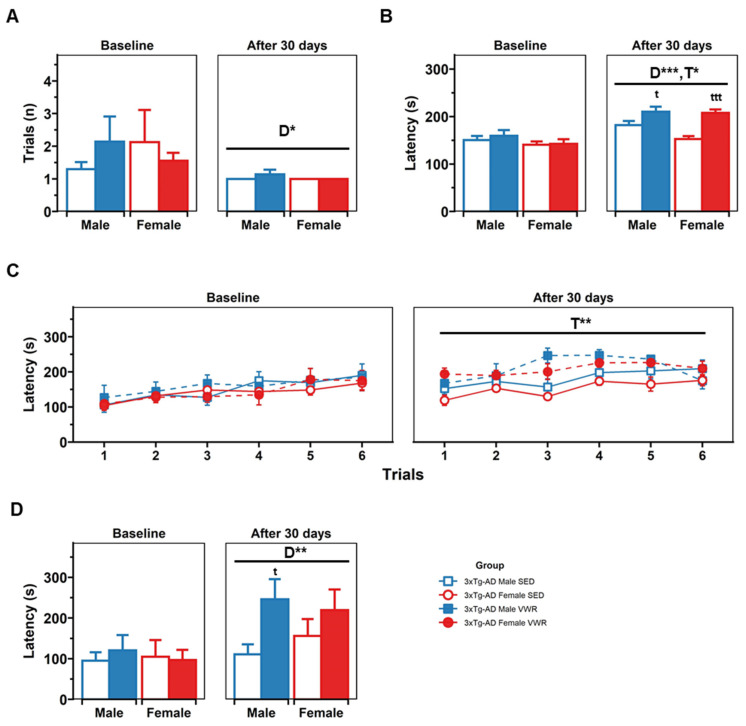
Rotarod test. (**A**) Learning phase. (**B**) Accelerated phase (pooled values). (**C**) Accelerated phase (trial by trial). (**D**) Rocking phase. Factorial analysis: D, days; T, treatment; S, sex. * *p* < 0.05; ** *p* < 0.01; *** *p* < 0.001. t < 0.05 and ttt < 0.001 vs. sedentary counterpart in the same sex.

In the constant mode of the rotarod (Figure 6), a days effect was found [F(1,30) = 6.10, *p* = 0.019, mixed ANOVA], with all animals learning faster when retested [t(34) = 2.43, *p* = 0.021, paired *t*-test]. Then, in the accelerated mode, a days effect [D, F(1,30) = 20.528, *p* < 0.001] and a treatment × days interaction [T × D, F(1,30) = 4.21, *p* = 0.049] effects were found. Further analysis showed that 3xTg-AD VWR males last longer than 3xTg-AD SED males (210.35 s vs. 182.1 s) [t(42,60) = −2.05, *p* = 0.04] and 3xTg-AD VWR females last longer than 3xTg-AD SED females (207.70 s vs. 152.83 s) [t(54,48) = −5.76, *p* < 0.001]. Then, when coordination was evaluated in the rocking mode, a general days effect was found [D, F(1,30) = 10.531, *p* = 0.003], in a post hoc analysis differences were found in the retest, with 3xTg-AD VWR males performing better than 3xTg-AD SED males [VWR vs. SED, t(10,7) = −2.44, *p* = 0.03, *t*-test].

### 2.5. Daily Patterns of Voluntary Wheel Running (VWR)

**Figure 7:**
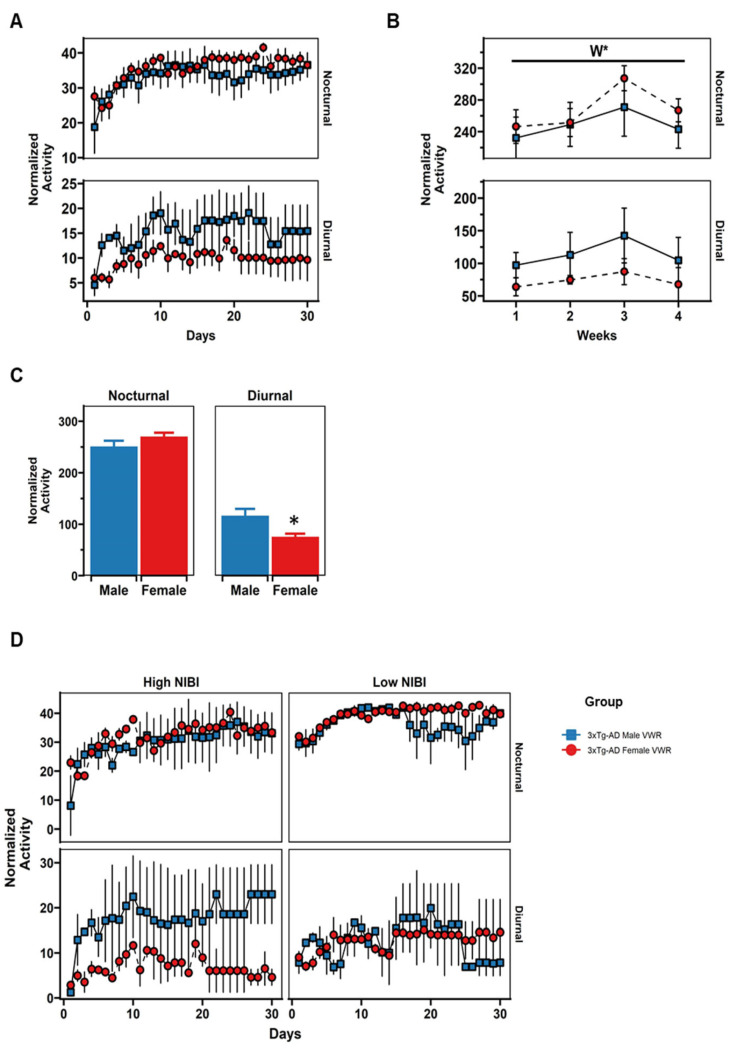
Patterns of voluntary wheel running (VWR). (**A**) Daily nocturnal (upper box) and diurnal (lower box) VWR activity. (**B**) Mean total activity in the RW week by week during nocturnal (upper box) and diurnal (lower box) periods. (**C**) Mean total activity per week during nocturnal and diurnal periods. (**D**) Activity in the RW adjusted by high and low novelty-induced behavioral inhibition (NIBI) in the corner test (CT) at baseline. W, weeks effect; * *p* < 0.05 and *t*-test vs. sex counterpart in the same period.

The pattern of behavioral circadian rhythmicity in the RW was evaluated in the animals allocated in the VWR group through two linear mixed effect models to assess the changes by sex in the trajectories of activity along 30 days and the influence of NIBI behavior in the CT at baseline (Figure 7A,D and Table 1). The main findings in model 1 were: (1) the average level of exercise increases with days in male cages during the nocturnal period (β_Days_: *p* < 0.001), (2) the difference in the average level of exercise between nocturnal and diurnal periods in male cages was significant at day 1 (β_Period_: *p* < 0.001), and (3) sex differences varies depending on the period at day 1 (β_Sex,Period_: *p* < 0.01); thus; VWR males performed less exercise than females in the first night of follow-up. Respecting model 2, the main findings showed that (1) the differences by sex were higher in the nocturnal period for high NIBI animals at day 1 (β_Sex,NIBI,Period_: *p* = 0.025) and (2) the slope of the VWR trajectory along nocturnal periods varies depending on the sex and the initial NIBI behavior (β_Days,Sex,NIBI_: *p* = 0.018).

In addition, a two-way ANOVA test was applied in the total levels of VWR pooled by weeks (Figure 7B). Here, a “Weeks” effect was found in the nocturnal period only [F(3,18) = 5.08, *p* = 0.01], explained by a significant decrease in the mean total activity by the fourth week compared to the third one [week 3 vs. week 4: t(8) = 4.40, *p* = 0.019, paired *t*-test].

When the mean total activity per week was considered (Figure 7C), significant sex differences were found during diurnal periods, with males performing higher level than females [males vs. females: t(16,16) = 2.30, *p* = 0.031, *t*-test].

Table 2 depicts the significant correlations in VWR group based on the aggregated values performed at baseline in the 8 cages equipped with the RW.

### 2.6. Survival

During the month of intervention, mortality rate was 5/39 (12.8%) and restricted to males (Chi-square = 4.432, 1 degrees of freedom, two-tailed *p* = 0.0353 vs. females), with 1/11 (9.09%) in the SED group and 4/11 (36.36%) in the VWR group (*n.s.*).

## 3. Discussion

An age-dependent decline in PA levels has been reported in healthy humans [34], and most of the species used in preclinical studies focusing on aging progression [21,22,35]. In clinical scenarios, PA engagement is found to decrease at higher rates in AD patients [17], pointing out the presence of NPS (i.e., apathy or depressive behavior) as a barrier to incorporating in recreational/social activities of higher energy consumption and further impeding the protective benefits from its regular practice [15]. Moreover, compared to healthy controls, AD patients show lower levels of PA during daylight periods, with a delay in the onset of their daily life activities during the morning, being more active during the night [8,17,36]. In addition, gender differences have been found in the circadian rhythms of movement activity in these patients, with males expending significantly more total energy than women during a 24 h period [18]. The causes of incremented activity overnight seem to be related to agitation or the “sundowning” condition in this population [37,38,39].

Here, we present for the first time the behavioral circadian rhythm pattern of PA in the animals submitted to VWR. Our main results showed sex-dependent differences in the mean total levels of VWR performed during diurnal periods. Thus, males remained more active than females during the daylight cycle.

Mice are nocturnal animals and their activity decreases during daylight hours [22]; therefore, we hypothesize this increment in energy expenditure during resting periods may be a sign related to hyperactive behavior, similar to that observed in the exacerbated agitation or “sundowning” behavior in humans. Interestingly, a second finding is that we also observed that the PA levels increased in the VWR animals along the days with a plateau after the third week of follow-up, attributable to a “learning phase” necessary for the animal’s adaptation or acclimation. A process previously reported occurring in rodent animal models under similar conditions of VWR [22,40,41,42].

In this proof-of-concept analysis of the animals in the VWR group, we found a mixture of meaningful behavioral correlations between the battery of tests performed at baseline and the VWR activity. Hence, in line with human studies [43], motor/body composition variables help predict the levels of PA performed during diurnal and nocturnal cycles. Specifically, better coordinated (according to the rocking phase of RR test) and stronger animals (based on grip 60 s results) positively correlated with total activity and mean nocturnal activity of the first 10 days of VWR follow-up. Furthermore, the weight at baseline positively correlated with the VWR activity. However, this correlation could be influenced by the sex differences found during the light-phase cycle (sexual dimorphism in body weight). On the other hand, variables of behavioral profiles such as neophobia in the CT and the exploratory activity in the OF test were also positively correlated with the VWR. Indeed, animals lasting longer to perform the first rearing in the CT (CTLaR) and to start the exploration in the OF central zone (Zcentral) run more in the wheel.

Interestingly, both linear mixed-effect models showed a significant sex-dependent difference in the VWR activity performed the first night of follow-up (day 1, nocturnal period) with male high-NIBI animals running less than females. These findings point out an influence of NPS-like symptoms (i.e., neophobia) on the circadian patterns of VWR of the 3xTg-AD animal model and/or different strategies to scope with the stress associated with novel environments. Nevertheless, further experiments are required to clarify the involvement and interaction of behavioral dimensions in VWR.

Taken together, the enhanced diurnal activity of males in the RW cannot be supported by sex motor differences since limb strength, endurance in the grip test, and coordination in the RR were identical for the SED and VWR groups at baseline and after 30 days. Therefore, we propose that there are factors beyond physical characteristics that influence the hyperactive diurnal pattern.

We have previously shown that at advanced stages of the disease, mortality rates are higher in 3xTg-AD males [44]. In the present work, sexual differences were also found with survival in all 17 females but mortality in 5 of the 22 males. In addition, the VWR male group showed a higher mortality ratio than the SED male group, albeit it did not reach statistical significance. This would agree with a previous work at the same stage of the disease, where only females survived in a VWR protocol, whereas NTg and 3xTg-AD males did not [45].

Considering all animals evaluated, an increase in neophobia with lower exploratory activity was detected in the CT after 30 days. These changes support a previous association of worsening NPS-like symptoms in the 3xTg-AD model, caused by the pathological aging process [46,47,48]. Furthermore, here, we tested the effect of PA starting at the late stages of AD, once cognitive and neuropsychiatric symptoms have been already established [29].

In our previous longitudinal study [49], including mice at 12 and 16 months of age at normal and AD-pathological stages, we found a reduction in the CTratio60 with age. Now, an increment in the CTratio60 was observed in the VWR group. We believe the increment was due to a general reduction in the CTrearings episodes, more than the potential beneficial effects of exercise. Thus, in experiments carried out in mice at earlier stages of AD [19], four weeks of voluntary running did not attenuate the reduction of horizontal (CTc60) or vertical activity (CTr60) in the test.

In the OF test, the exploratory activity is commonly used to analyze the animal’s response to stressful/anxiogenic events [50]. Indeed, we have shown that in the 3xTg-AD mice the usage of repeated of tests can help monitor the development of AD [51]. Interestingly, in a study with 20 different mouse strains [52], a positive correlation between the distance covered on the test and the distance run in several days of VRW follow-up has been described. However, in the present study, we found no correlation between such variables.

Animals submitted to VWR reduced the latency to move (Movement) and to start exploration (Zcentral) in the OF, indicating a possible reduction in the test-induced anxiety or an improvement in the habituation process to the test, which is also one of the simplest types of memory [48].

The behavior of all animals in the TMSA was similar, with most failing to achieve the completion criteria of the test. This can be related to the advanced aged status of the animals and lack of motivation [44]. Moreover, differences in TMSA performance have been linked to impaired immune function and shorter lifespans in slower animals [53]. Here, the 3xTg-AD Male VWR group tended to complete the exploratory goals in a shorter time. Indeed, they crossed the intersection of the maze significantly faster than all other groups. This would agree with their faster performance in exploratory variables in the OF test mentioned above.

Improvement in muscular strength and endurance in the VWR group are presumably associated with the benefits of exercise. Nevertheless, sedentary animals also improved after 30 days. This increment can be related to the effect of a previous experience because the animals performed better when reassessed [48]. Another factor affecting the increase in forelimb strength involves the natural activities of the animals in their home cages, such as grid climbing [54]. Because the RW, as an object, may represent an enrichment in the environment for the VWR group, the optimal SED groups should include such an environmental cage condition (a blocked wheel). Still, in previous experiments [19,45] using this kind of SED control group, we have demonstrated that the benefits of the exercise can be clearly discriminated compared to those 3xTg-AD mice housed in a cage with a blocked wheel.

Finally, in the RR test, VWR animals outperformed the SED group in the accelerated and coordination protocols, probably due to muscle memory and the similarity of RR apparatus and the RW used as treatment.

In summary, the main findings of the present work are: (1) The 3xTg-AD male model seems to replicate some physical activity circadian rhythm dysfunctions, with enhanced diurnal activity compared to females. (2) The increased activity in males seems to be associated with NPS-like symptoms. (3) Despite advanced age and AD pathology, VWR improved some anxiety-related NPS-like symptoms, such as freezing behavior in the OF and TM. However, cognitive improvement was not observed. These findings support the importance of tailored and individualized physical activity programs in clinical settings (walking, daily life activities) that should be designed and implemented considering sex/gender, NPS profile, and circadian rhythms.

## 4. Methods

### 4.1. Animals

The 3xTg-AD model was previously generated [25] by microinjecting (1) a human cDNA harboring the APP transgene, with a translocation in amino acids, named the Sweden mutation (APPSWE), and (2) the human P301L tau transgene (tauP301L), which impairs binding of tau from microtubules, into single-cell embryos of homozygous knockin mice (hybrid 129/C57BL6 background), with mutations in presenilin 1 (PS1M146V), and a protease forming one of the domains of the β-secretase protein.

An initial sample of thirty-nine 14-months-old 3xTg-AD animals (22 males and 17 females) from the Spanish colony of homozygous 3xTg-AD mice, established at the Autonomous University of Barcelona, were included in the study and assigned to the sedentary (SED: 11 males and 8 females) or voluntary wheel-running (VWR: 11 males and 9 females) group in a counterbalanced manner after considering the number of visited corners in the corner test at 60 s (CTc60) in the baseline assessment.

All animals were kept in 12 h light–dark cycles starting at 8 a.m., with vivarium temperature at 22 ± 2 °C and free access to food and water.

During the month of intervention, the mortality rate was monitored daily. The data of animals completing the 30 days of intervention were gathered and analyzed with the following animals per group composition: thirty-four animals (17 males and 17 females) allocated in the sedentary (SED: 10 males and 8 females) and voluntary wheel running (VWR: 7 males and 9 females) groups.

### 4.2. Behavioral Assessment

#### 4.2.1. Corner Test (CT)

Neophobia response was evaluated in the CT by direct observation of the animal’s behavior in a new home cage for 1 min. The test started with the mouse in the center of the cage; then, the horizontal (number of corners at 30 s and 60 s and number of corners until the first rearing) and vertical (number of rearings at 30 and 60 s) activity were recorded. The ratio of these variables was also calculated (ratio corners/rearings at 30 s and 60 s).

#### 4.2.2. Open Field (OF) Test

The exploratory activity was evaluated in an OF (metalwork, beige, 44 × 38 × 10 cm height) for 5 min. Animals were placed in the central area, and vertical (number and latency of rearings and groomings) and horizontal (latencies until the first movement and reaching the periphery) behavior was registered. In addition, the total distance was recorded using the VideoTrack analysis system (ViewPoint Behavior Technology, Lyon, France). Finally, the number of defecation boli and urination episodes were also noted.

#### 4.2.3. T-Maze Spontaneous Alternation (TMSA)

The spontaneous alternation task paradigm in the T-shaped maze (two short arms of 30 × 10 cm^2^ and a single long arm of 50 × 10 cm^2^) was used to evaluate coping with stress strategies, risk assessment, and working memory. Thus, animals started the test facing the end wall of the long arm and were free to explore for a maximum of 5 min. Latencies of horizontal activity involved the time until the first move and turn, reaching the intersection of the three arms, crossing the intersection with their 4 paws, and exploring the two short arms (test completion criteria). Latencies of vertical activity comprised the number of rearings and groomings. Defecation and urination were considered too.

#### 4.2.4. Sensorimotor Assessment

The muscle strength of the animals was evaluated in the muscular strength (grip 5 s) and muscular endurance (grip 60 s) paradigms of the grip test. Here, two trials (5 s and 60 s) were carried out, and the duration of grasping a bar using their forepaws while suspended was registered. Next, response to gravity was assessed in a single trial in the 90° Geotaxis test. Thus, animals were placed in a grid (10 cm × 12 cm) facing downwards and the latency until reaching a vertical position (facing upwards) was recorded.

#### 4.2.5. Rotarod

The constant, accelerated, and rocking protocols of rotarod (Ugo Basile, mouse rotarod NG) were utilized. The apparatus consists of five rods (3 cm diameter) located at 16 cm height, and six 25 cm dividers forming five lanes, where animals are placed with their back to the experimenter. Steel “Trip boxes” are placed below each rod to receive mice after falling off.

Firstly, in the constant mode, mice run over the rods at a continuous 10 rpm and the number of trials needed to reach 60 s without falling was reported. A maximum of 10 trials were performed.

Then, the accelerated mode was configured to an acceleration of 0–48 rpm for six trials with a maximum of 6 min each (resting time between trials: 1 min). A trial was finished when the animal fell from the rod.

Finally, in the single rocking mode, coordination was assessed by changing the direction of rod rotation after reaching 20 rpm.

### 4.3. Intervention Protocol

Mice in the VWR group were housed in groups of 2–3 in cages equipped with an RW (36 × 20 × 14 cm, activity wheel cage system for mice, Techniplast, Buguggiate, Italy). Those in the SED group were in similar cages without RW.

VWR 3xTg-AD mice were free to perform voluntary PA for 30 continuous days. The system allowed assessment of circadian motor activity by recording revolutions on the wheel, which were registered at 8 h (Nocturnal activity) and 20 h (Diurnal activity).

### 4.4. Statistical Analysis

Most behavioral data were analyzed using mixed models analyses of variance (ANOVAs), with sex and treatment as between factors, and days as within factor. Pairwise *t*-test comparisons were used as a post hoc method.

In the circadian behavioral analysis of VWR, a linear mixed-effect model was used to calculate the association of VWR activity in two different models. In Model 1, the “fixed effect” comprised the interaction between a dependent variable (normalized VWR activity) and independent variables days (30 days of follow-up), sex (male and female) and periods (diurnal and nocturnal). In Model 2, normalized VWR activity was adjusted by the previous performance of the animals in the CT. Thus, animals were classified as presenting high (below the 33rd percentile in the CTc60) or low (above the 33rd percentile in CTc60) novelty-induced behavioral inhibition (NIBI) [30].

Results were expressed as the mean ± standard error of the mean (SEM). All the analyses were made using the open-source programming language R software, version 4.1.3.

## Figures and Tables

**Table 1:** Results for the linear mixed-effects models assessing the activity in the RW by days (Model 1) and adjusted by previous NIBI behavior in the CT (Model 2).

Variable	Estimate	SE	Z Value	*p*-Value
Model 1	Revolutions~I (Days-1) ×Sex × Period + (1|Cage)			
β_0_	29.71	2.68	11.09	<0.001 (***)
β_Days_	0.24	0.06	4.05	<0.001 (***)
β_Sex_	1.11	3.79	0.29	*n.s.*
β_Period_	−16.59	1.40	−11.81	<0.001 (***)
β_Days,Sex_	0.10	0.08	1.20	*n.s.*
β_Days,Period_	−0.09	0.08	−1.12	*n.s.*
β_Sex,Period_	−5.61	1.99	−2.82	0.004 (**)
β_Days,Sex,Period_	−0.17	0.12	−1.41	*n.s.*
Model 2	Revolutions~I (Days-1) × Sex × NIBI × Period + (1|Cage)			
β_0_	22.97	3.83	5.99	<0.001 (***)
β_Days_	0.48	0.07	6.47	<0.001 (***)
β_Sex_	3.12	5.42	0.58	*n.s.*
β_NIBI_	13.48	5.42	2.49	0.013 (*)
β_Period_	−9.57	1.78	−5.38	<0.001 (***)
β_Days,Sex_	−0.08	0.11	−0.73	*n.s.*
β_Days,NIBI_	−0.49	0.11	−4.62	<0.001 (***)
β_Sex,NIBI_	−4.02	7.67	−0.52	*n.s.*
β_Days,Period_	−0.17	0.11	−1.63	*n.s.*
β_Sex,Period_	−9.59	2.52	−3.81	<0.001 (***)
β_NIBI,Period_	−14.04	2.52	−5.58	<0.001 (***)
β_Days,Sex,NIBI_	0.35	0.15	2.38	0.018 (*)
β_Days,Sex,Period_	−0.24	0.15	−1.60	*n.s.*
β_Days,NIBI,Period_	0.16	0.15	1.05	*n.s.*
β_Sex,NIBI,Period_	7.96	3.56	2.24	0.025 (*)
β_Days,Sex,NIBI,Period_	0.15	0.21	0.69	*n.s.*

*p*-Value significance level: * *p* < 0.05; ** *p* < 0.01; *** *p* < 0.0.01.

**Table 2:** Significant correlations in VWR group (*n* = 8 cages).

	Rearing Latency (CT)	Center Latency (OF)	Total Grooming (OF)	Rocking (RR)	Grip 60	Initial Weight
Total activity (in 30 days)	0.511	0.737 *	0.669	0.719 *	0.371	0.712 *
Mean nocturnal activity	0.354	0.583	0.714 *	0.635	0.482	0.746 *
Mean diurnal activity	0.745 *	0.644	−0.101	0.557	0.007	0.308
Mean daily activity	0.491	0.681	0.642	0.711 *	0.450	0.760 *
Mean nocturnal activity (days 1–10)	−0.114	0.229	0.605	0.101	0.721 *	0.730 *
Mean diurnal activity (days 1–10)	0.781 *	0.592	−0.013	0.688	−0.038	0.215
Ratio days 1–10	−0.800 *	−0.174	−0.582	−0.674	−0.048	−0.051

Data showing Pearson’s correlation between variables of VWR and the initial battery of tests (CT, OF, RR, sensorimotor assessment, and initial body weight) of animals in the VWR group. * *p* < 0.05.

## Data Availability

Not applicable.
